# The influence of the COVID-19 epidemic on hospitalizations due to alcohol consumption

**DOI:** 10.1192/j.eurpsy.2021.1512

**Published:** 2021-08-13

**Authors:** I.D. Rădulescu, M. Terpan, A. Ciubară

**Affiliations:** 1 Psychiatrist, “Elisabeta Doamna” Psychiatric Hospital, Galati, Romania; 2 Corresponding Author, Ph.d Student, “Dunarea de Jos“ University, Galati, Romania; 3 Md, Ph.d., Hab. Professor, Faculty of Medicine and Pharmacy, University “Dunarea de Jos” Head of Psychiatry Department, Senior Psychiatrist at ”Elisabeta Doamna” Hospital, Galati, Romania

**Keywords:** Acut Intoxication, Alchohol, pandemic, Addictive disorders

## Abstract

**Introduction:**

Excessive alcohol consumption is an ever-topical issue regardless of social or medical problems (pandemic). In these conditions (global medical crisis),to the problem of alcohol consumption has been added a new dimension.

**Objectives:**

The main purpose of this study is to analyze the impact of the COVID-19 pandemic on hospitalizations diagnosed with acute intoxication in the hospital. In Romania, the measures due to the pandemic were instituted starting with March 15 2020.

**Methods:**

The study was performed retrospectively between 01.01.2020 - 30.09.2020 in the Psychiatric Hospital ‘Elisabeta Doamna’ Galati. ICD-10 criteria were used to establish the diagnosis of the disorder.

**Results:**

In total, 458 cases were admitted during the period mentioned, of which 401 were male (87.56%), female 57 cases (12.44%). The average age of patients was 45.67 years ± 0.695, with minimum age of 19 years and maximum age of 93 years. The month with the most admissions was January with 80 (17.46%) March by 79 (17.25 %). The months with the fewest hospitalizations were April with 27 cases (5.89%) and July with 35 cases (7.64%).

**Conclusions:**

The analysis of the data shows that as measures specific to the epidemic crisis were instituted, the number of hospitalizations decreased significantly by about 3 times.

